# Draft Genome Sequence of Thermophilic Halotolerant Aeribacillus pallidus TD1, Isolated from Tao Dam Hot Spring, Thailand

**DOI:** 10.1128/MRA.00204-19

**Published:** 2019-04-25

**Authors:** Wariya Yamprayoonswat, Satapanawat Sittihan, Watthanachai Jumpathong, Montri Yasawong

**Affiliations:** a Environmental Toxicology, Chulabhorn Graduate Institute, Bangkok, Thailand; b Center of Excellence on Environmental Health and Toxicology (EHT), Office of Higher Education, Bangkok, Thailand; c Chemical Biology, Chulabhorn Graduate Institute, Bangkok, Thailand; University of Arizona

## Abstract

Aeribacillus pallidus TD1 is a thermophilic bacterium isolated from a hot spring in Thailand. The genome sequence of *A. pallidus* TD1 contains a gene-encoded naphthalene dioxygenase, which is a key enzyme for naphthalene degradation. This 3.7-Mb draft genome sequence of *A. pallidus* TD1 will contribute to the understanding of polycyclic aromatic hydrocarbon (PAH) degradation in high-temperature environments.

## Abstract (precis)

Aeribacillus pallidus TD1 is a thermophilic bacterium isolated from a hot spring in Thailand. The genome sequence of *A. pallidus* TD1 contains a gene-encoded naphthalene dioxygenase, which is a key enzyme for naphthalene degradation.

## ANNOUNCEMENT

Aeribacillus pallidus TD1 was isolated from Tao Dam hot spring in Khluang Wangchao National Park, Thailand (1). The 16S rRNA gene of the strain TD1 was 99% identical to that of Aeribacillus pallidus DSM 3670 (1). The strain TD1 is a Gram-positive, rod-shaped, and aerobic bacterium. Cells are motile and endospore forming (1). The optimum growth occurred at 55 to 60°C and pH 7 to 8. The strain TD1 is halotolerant because it was able to grow on medium containing up to 10% NaCl (1). The major fatty acid component of *A. pallidus* TD1 was C_16:0_ (1).

The bacterial strain TD1 (DSM 22570) was obtained from The Leibniz Institute DSMZ-German Collection of Microorganisms and Cell Cultures. It was grown in R2A broth at 55°C overnight with a shaking speed of 250 rpm. Genomic DNA (gDNA) of *A. pallidus* TD1 was obtained with the NucleoSpin microbial DNA isolation kit (Macherey-Nagel, Germany). NanoDrop spectrophotometry (Thermo Fisher Scientific, USA) was used to quantify the concentration of gDNA. A sequencing library was prepared with the Ion PI Hi-Q OT2 template kit (Thermo Fisher Scientific) and Ion Plus fragment library kit (Thermo Fisher Scientific). The library was placed on an Ion PI chip kit v3 (Thermo Fisher Scientific), and the sequencing was performed with an Ion Proton sequencer (Thermo Fisher Scientific). The sequencing run generated 12,033,229 raw reads (303× depth of coverage), with an average length of 115 bp from the sequencing.

*De novo* genome assembly was performed with the raw reads and SPAdes 3.13.0 in the –careful mode (2). The genome assembly metric was calculated with QUAST v5.0.2 using default parameters (3). The draft genome sequence of *A. pallidus* TD1 consists of 3,736,803 bp in 167 contigs with an *N*_50_ value of 48,997 bp and a GC content of 38.8%. Gene prediction was performed with Prokka v1.13.3 using default parameters (4). The annotated genome sequence of *A. pallidus* TD1 contains 3,600 protein-coding sequences along with 50 tRNA genes, 3 rRNA genes, and 1 transfer-messenger RNA (tmRNA) gene.

One annotated locus encodes the *ndoA* gene in the *A. pallidus* TD1 genome. The *ndoA* gene is encoded naphthalene dioxygenase, which is a key enzyme for naphthalene degradation (5). This enzyme adds two oxygen atoms to the aromatic ring of polycyclic aromatic hydrocarbons (PAHs) to form the corresponding *cis*-dihydrodiol derivatives (5).

### Data availability.

The whole-genome shotgun sequence of Aeribacillus pallidus TD1 has been deposited at DDBJ/ENA/GenBank under the accession number SFCD00000000 and SRA accession number SRR8543811.
